# The MurG glycosyltransferase provides an oligomeric scaffold for the cytoplasmic steps of peptidoglycan biosynthesis in the human pathogen *Bordetella pertussis*

**DOI:** 10.1038/s41598-019-40966-z

**Published:** 2019-03-15

**Authors:** Federica Laddomada, Mayara M. Miyachiro, Matthew Jessop, Delphine Patin, Viviana Job, Dominique Mengin-Lecreulx, Aline Le Roy, Christine Ebel, Cécile Breyton, Irina Gutsche, Andréa Dessen

**Affiliations:** 1 Univ. Grenoble Alpes, CNRS, CEA, Institut de Biologie Structurale (IBS), F- 38000 Grenoble, France; 2 Brazilian Biosciences National Laboratory (LNBio), CNPEM, Campinas, 13084-971 São Paulo Brazil; 3 Universidade Estadual de Campinas (UNICAMP), Departamento de Genética, Evolução e Bioagentes, Campinas, São Paulo Brazil; 4 Institute for Integrative Biology of the Cell (I2BC), CEA, CNRS, Univ Paris-Sud and Université Paris-Saclay, 91198 Gif-sur-Yvette, France

## Abstract

Peptidoglycan is a major component of the bacterial cell wall and thus a major determinant of cell shape. Its biosynthesis is initiated by several sequential reactions catalyzed by cytoplasmic Mur enzymes. Mur ligases (MurC, -D, -E, and -F) are essential for bacteria, metabolize molecules not present in eukaryotes, and are structurally and biochemically tractable. However, although many Mur inhibitors have been developed, few have shown promising antibacterial activity, prompting the hypothesis that within the cytoplasm, Mur enzymes could exist as a complex whose architecture limits access of small molecules to their active sites. This suggestion is supported by the observation that in many bacteria, *mur* genes are present in a single operon, and pairs of these genes often are fused to generate a single polypeptide. Here, we explored this genetic arrangement in the human pathogen *Bordetella pertussis* and show that MurE and MurF are expressed as a single, bifunctional protein. EM, small angle X-ray scattering (SAXS), and analytical centrifugation (AUC) revealed that the MurE–MurF fusion displays an elongated, flexible structure that can dimerize. Moreover, MurE–MurF interacted with the peripheral glycosyltransferase MurG, which formed discrete oligomers resembling 4- or 5-armed stars in EM images. The oligomeric structure of MurG may allow it to play a *bona fide* scaffolding role for a potential Mur complex, facilitating the efficient conveyance of peptidoglycan-building blocks toward the inner membrane leaflet. Our findings shed light on the structural determinants of a peptidoglycan formation complex involving Mur enzymes in bacterial cell wall formation.

## Introduction

**Figure 1:**
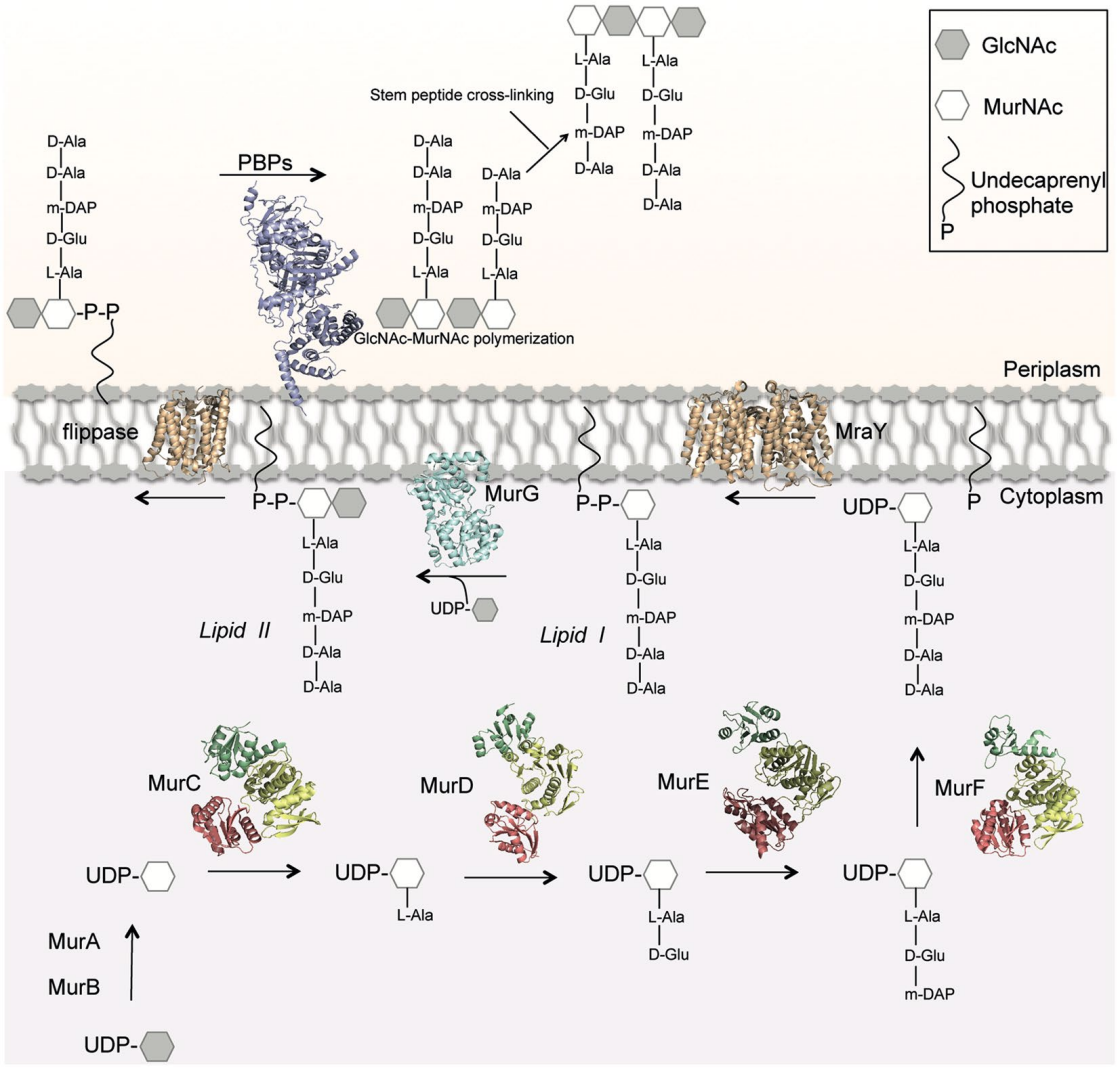
Schematic diagram of the cytoplasmic and membrane-related steps of peptidoglycan biosynthesis. The scheme includes structures of macromolecules from different bacterial species: MurA (1NAW); MurB (1MBT); MurC (1J6U); MurD (4BUC); MurE (4BUB); MurF (3ZL8); MurG (1F0K); MraY (4J72).

The bacterial cell wall is a complex structure that plays key roles in cell shape and maintenance of osmotic pressure. One of the main components of the cell wall, the peptidoglycan, is a three-dimensional mesh that envelopes the entire bacterial cell and is formed by polymerized chains of repeating disaccharide subunits (GlcNAc and MurNAc) cross-linked by stem peptides^1,2^. Three cellular compartments are involved in peptidoglycan biosynthesis (cytoplasm, membrane, and periplasm). Reactions that occur within the cytoplasm involve the formation of a soluble precursor (UDP-MurNAc-pentapeptide, or UM-pentapeptide) and its association to the inner leaflet of the membrane through MraY^3^. This links the P-MurNAc-peptide motif onto a C55-P (undecaprenyl phosphate) carrier lipid. Subsequently, the glycosyltransferase MurG associates a GlcNAc moiety to Lipid I, generating Lipid II, which is then translocated towards the periplasmic space by flippases^4,5^. In the periplasm, Penicillin-Binding Proteins (PBPs) catalyze the two last reactions in peptidoglycan biosynthesis (GlcNAc-MurNAc polymerization, or transglycosylation, and stem peptide cross-linking, or transpeptidation; Fig. 1)^2^. Recently, proteins from the SEDS (Shape, Elongation, Division, and Sporulation) family were also reported to catalyze glycan chain polymerization in some species^6^, often in partnership with PBPs^7,8^.

Proteins that are involved in peptidoglycan biosynthesis have been shown to associate in discrete multi-membered complexes, namely the ‘divisome’, that regulates cell division, and the elongasome, or Rod complex, that is involved in lateral wall formation in rod-shaped cells^9^. The reactions catalyzed within the cytoplasm are common to these two processes. Subsequent to the generation of UM (UDP-MurNAc) by the concerted action of MurA and MurB enzymes, a family of ATP-dependent enzymes, MurC, D, E, and F catalyze the stepwise ligation of amino acids onto UM (Fig. 1). MurC adds an L-alanine group, forming UDP-*N*-acetylmuramoyl-L-alanine (UMA), and this is followed by the addition of D-glutamate by MurD. The subsequent reaction is catalyzed by MurE, and involves the association of either *meso*-diaminopimelate (A_2_pm or DAP) or L-lysine, depending on the species^10^. MurF, the last enzyme in this section of the pathway, catalyzes the addition of the D-alanyl-D-alanine dipeptide to the UM-tripeptide, thus generating UM-pentapeptide^11^.

Mur ligases (MurC-MurF) display highly similar 3D structures, with an N-terminal domain which binds the UM, UMA, or UM-peptide precursors, a central ATP-recognizing domain, and a C-terminal region that binds the amino acid(s) to be added onto the precursor^11^. These enzymes have been extensively characterized in isolated form, but information regarding the coordination of their activities and potential partner recognition has remained elusive. Notably, in organisms such as *Bordetella* and *Pseudovibrio* spp., as well as in certain species of archaea, MurE and MurF are encoded as a single polypeptide. Since MurE and MurF catalyze two subsequent steps in Lipid I biosynthesis, this could suggest that the UM-tripeptide intermediate could be shuttled between the two active sites in the fused protein, providing a catalytic advantage for the cell. Similar observations have been made with MurB and MurC, MurG and MurC, MurC and Ddl, and MraY and MurG^12^. Interestingly, some of these fused proteins are encoded by genes that do not catalyze subsequent steps in the peptidoglycan pathway (such as MurG/MurC and MurC/Ddl)^13,14^, which brings up questions regarding a catalytic advantage for the cell in their association while still underlining the potential relevance of a multi-protein complex.

The potential existence of a cytoplasmic complex involving Mur enzymes has been suggested by different laboratories^15,16^. White and co-workers showed that MurB, MurC, MurE, MurF and MraY all localize in *Caulobacter crescentus* in a manner that is similar to that of MurG^15^. In *E. coli*, MurG was also shown to interact with both MraY and the cytoskeletal protein MreB, suggested as being an organizing agent for cell wall elongation^17^. Subsequently, MurG was also shown to bind MurD, MurE, and MurF ligases from the thermostable bacterium *Thermotoga maritima*^18^. These observations suggest that MurG could act as a scaffold for Mur enzymes, aiding in the restriction of the diffusion of soluble peptidoglycan biosynthesis intermediates within the cytoplasm and directing them towards the internal side of the membrane^15^. However, the structural nature of such a scaffold has not been studied to date.

In this work, we characterized the scaffolding role of MurG from the human pathogen *Bordetella pertussis*, the causative agent of whooping cough, by examining its oligomeric state and stoichiometry both *in vitro* and on bacterial membranes. MurG is dimeric in the presence of detergents, but oligomerizes into higher order species (tetramers and above) in their absence. Negative staining electron microscopy (EM) images of MurG oligomers revealed isolated particles that resemble 4- or 5- pointed stars. In *B. pertussis*, MurE and MurF are expressed as a single, fused polypeptide, and the different forms of MurG recognize MurE-MurF with similar affinities. These results shed light onto the structural determinants of a peptidoglycan formation complex involving Mur enzymes that plays a role in cell wall formation in a pathogenic species, but that can be applicable to all walled bacteria.

## Results

### MurG form oligomers with different stoichiometries

MurG is a peripheral membrane protein that interacts with phospholipids of the cytoplasmic membrane^17,19,20^. The crystal structure of the *E. coli* enzyme suggests that this interaction occurs through an N-terminal hydrophobic patch that is surrounded by basic residues^21^. MurG has been reported to behave as a dimer^22^, but to date the structural determinants and functional significance of this arrangement have not been reported. Given the fact that MurG has been reported to serve as a potential scaffold for other peptidoglycan biosynthesis enzymes and that this function could be impacted by its oligomeric form, we set out to characterize MurG both *in vitro* and *in cellulo*.

**Figure 2:**
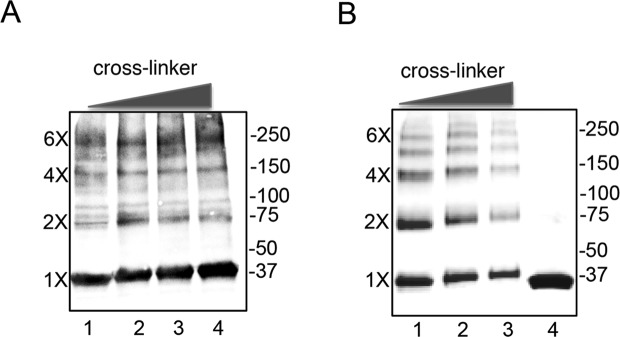
MurG adopts different oligomeric states in bacterial membranes and *in vitro*. (**A**) Western Blot of MurG present in purified *E. coli* membranes treated with increasing concentrations of DMP (lanes 1–4). Molecular weight markers are shown to the right of the blot and the approximate location of monomers (1X) to hexamers (6X) is shown on the left. (**B**) SDS-PAGE of MurG overexpressed and purified as a soluble protein in presence of detergent was treated with increasing concentrations of EGS (ethylene glycol bis(succinimidyl succinate)). Lanes 1–3 show samples treated with 0.1, 1.0, and 2.5 mM EGS, respectively. Lane 4 purified, untreated MurG.

In order to characterize the oligomeric state of MurG on bacterial membranes, we expressed the 357-residue, N-terminal strep-tagged MurG from *B. pertussis* in *E. coli*, isolated and purified inner membranes, and cross-linked the sample with dimethyl pimelimidate (DMP). The protein content of the membrane was evaluated by SDS-PAGE and Western blotting using anti-MurG antibodies (Fig. 2A). Despite the fact that monomeric MurG seemed to be the most abundant form, a range of higher oligomeric states was also observed that could correspond to dimers, tetramers, or even hexamers. Interestingly, when the cross-linking experiment was performed with the purified form of MurG, the same oligomeric forms were also identified (Fig. 2B). These data demonstrated that MurG forms oligomers both *in vitro* and within membranes, and thus oligomerization could have an impact on MurG functionality in the cell.

**Figure 3:**
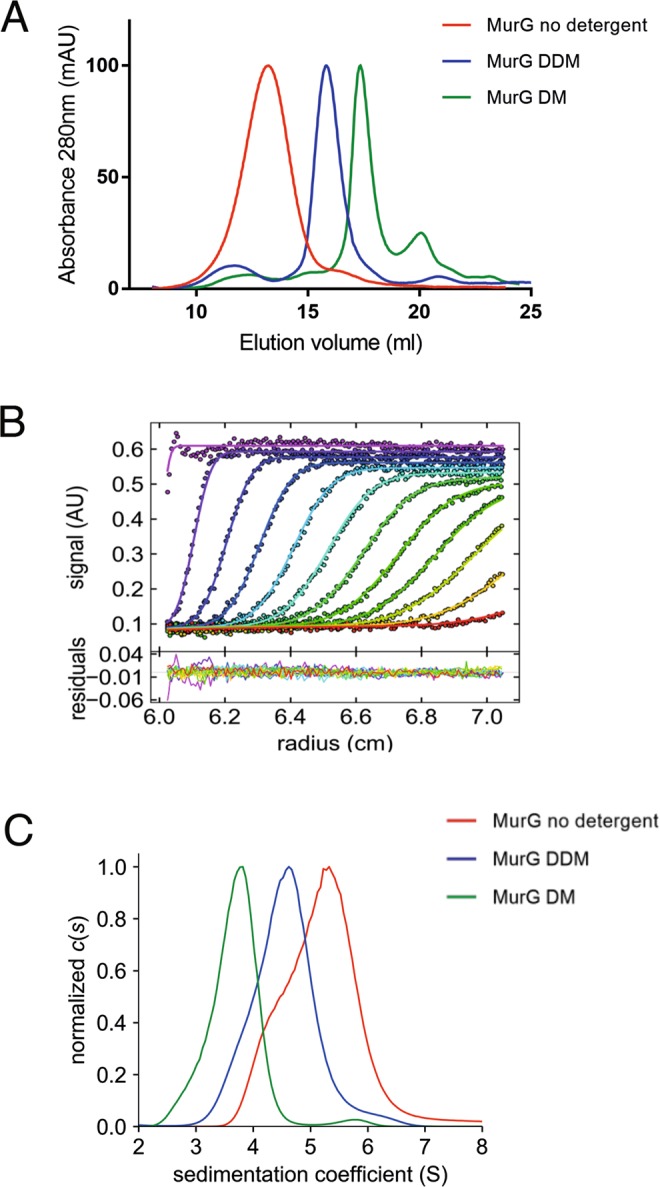
Biophysical characterization of purified MurG. (**A**) SEC analysis of MurG performed on a Superose 6 10/300 column. DDM and DM were used at 1.2 x CMC. (**B**) Analytical ultracentrifugation sedimentation profiles of MurG in buffer containing DDM: experimental and fitted values (top panel) and residuals (lower panel). The profiles correspond to absorbance measurements at 250 nm made in buffer containing DDM during 8 h of sedimentation, at 42,000 rpm and 20 °C. Data obtained in interference (J) and absorbance at 280 nm are not shown for simplicity. (**C**) Corresponding *c*(*s*) distributions of MurG in buffer without detergents, or in the presence of DM or DDM (1.2 x CMC).

In order to further characterize these oligomers, we analyzed purified MurG by size exclusion chromatography (SEC) either in the presence of detergents (DDM (n-dodecyl-β-D-maltoside) and DM (decyl-β-D-maltoside) at 1.2 x CMC) or in their absence. When the experiment was performed in detergent-free buffer, MurG eluted as a broad peak at the beginning of the elution profile, suggesting a mixture of large oligomeric species (Fig. 3A). However, in the presence of either DDM or DM, MurG eluted later, in distinct peaks that could suggest monomers or dimers. These results indicated that MurG can vary its quaternary state depending on the conditions employed.

Analytical ultracentrifugation (AUC) was thus performed on MurG in the three conditions described above. Consecutive scans were automatically recorded at regular intervals and analyzed by Sedfit using a continuous size distribution *c(s)* analysis to determine the sedimentation coefficients, *s*. In all three experiments, MurG was tested at a concentration of 2 mg/ml. The interpretation of the *s*-values was done taking into consideration the frictional ratio, *f/f*_min_, which is reflective of molecular shape; i.e., a value of 1.25 indicates a globular compact species^23^. In experiments performed with detergent, the amount of bound detergent was experimentally derived from sedimentation data obtained using interference optics and performing measurements at 280 nm. Data are reported in Figs. 3B,C and Table 1.

**Table 1:** Sedimentation velocity AUC results for MurG purified in the presence or absence of detergents.

Sample	*s* (S)	*s*_20w_ (S)	Potential main oligomeric form	*f*/*f*_min_	δ (g/g)
MurG	5.10 +/− 0.05	7.2 +/− 0.10	Mixture of globular trimer and tetramer/elongated tetramer	1.25/1.4	0/0
MurG+DDM	4.50 +/− 0.05	6.5 +/− 0.05	Globular dimer/elongated trimer	1.24/1.60	0.8/0.8
MurG+DM	3.57 +/− 0.08	5.5 +/− 0.15	Globular/elongated dimer	1.25/1.55	1.4/0.8

The *s*-value corresponds to the main contribution in the *c*(*s*) curve; *s*_*20,w*_ is the *s*-value transformed to standard conditions (water at 20 °C); the potential oligomeric forms were determined considering different frictional ratios *f*/*f*_min_. δ represents bound detergent, in g/g. The value δ = 0.8 g/g was experimentally derived in DDM.

When MurG was purified in DDM, its sedimentation profile yielded a peak at *s* = 4.5 +/− 0.05 S (*s*_*20,w*_ = 6.5 S +/− 0.05 S), that could correspond to a compact dimer (f/f_min_ = 1.25) or an elongated trimer (*f*/*f*_min_ = 1.4) that binds 0.8 g/g of DDM (the latter value having been experimentally derived). In DM, the sedimentation profile of MurG showed a major peak at s of 3.57 +/− 0.08 S (_*s20,w*_ = 5.5 +/− 0.15 S). Even though the amount of bound detergent could not be estimated because the contributions of free micelles and MurG could not be clearly separated, the *s*-value reasonably corresponds to a dimer (Table 1). In the absence of detergent, however, we observed a large asymmetrical main peak with a max *s-*value of 5.1 +/− 0.05 S (*s*_*20,w*_ of 7.24 S +/− 0.1 S). The broad distribution of s values suggests a mixture of different oligomers, which could include a tetramer as well as forms of lower and higher molecular mass.

### MurG oligomers form individual star-like particles

Our cross-linking analyses of MurG in bacterial membranes indicated that higher oligomeric forms are present (Fig. 2A) and could play a role in its functionality. We thus set out to characterize the detergent-free, larger oligomeric forms of MurG by negative staining EM. MurG was purified from *E. coli* membranes with detergent and gel filtered in a buffer that did not contain detergents. Subsequently, the purified sample was subjected to GraFix fixation^24^ on a gradient of 0–0.15% glutaraldehyde and 10–30% glycerol. After ultracentrifugation, the gradient tubes were fractionated and different fractions were observed by negative stain EM.

**Figure 4:**
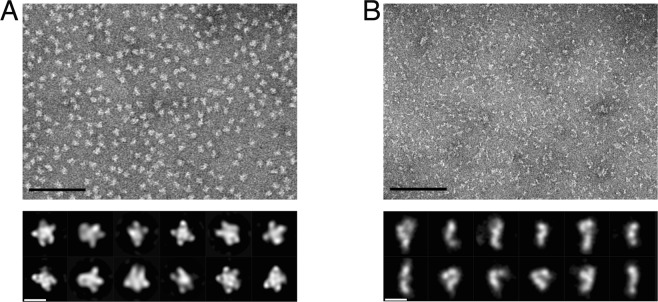
Negative-stain electron microscopy of MurG and MurE-MurF samples. (**A**) Representative micrographs of MurG and (**B**) MurE-MurF, scale bar = 100 nm. Below: gallery of *ab initio* class averages of MurG (left) and MurE-MurF (right), scale bar = 10 nm.

Electron micrographs (Fig. 4A) show compact, semi-homogeneous structures with diameters in the range of 100 × 150 Å. Visual inspection of the images made us think that the preparation might be homogeneous enough to allow a low-resolution 3-D analysis. 2-D classification of circa 14,000 individual frames resulted in well-defined class-averages with prominent “star-like” features with 4 or 5 arms. Yet, attempts to calculate a 3-D model were unsuccessful, suggesting that MurG was not present in one distinct state and pointing to structural heterogeneity and/or conformational flexibility of the MurG oligomers. Most importantly, class-averages did however indicate that MurG can form discrete oligomers that are distinct from monomers or dimers suggested by crystal structure data. It is of note that the crystal structure of MurG from *E. coli*, that displays two molecules related by a two-fold axis in the asymmetric unit, was obtained in the presence of Triton X-100^21,25^, and this arrangement differs considerably from what we imaged here.

The capability to form tetramers or higher order species as well as the presence of flexible regions could play a role in the ability of MurG to serve as a framework for other Mur ligases. Thus, in order to expand our study of a Mur ligase complex scaffolded by MurG, we set out to characterize a potential interaction partner, the MurE-MurF fusion protein, as well as the interaction between the different Mur enzymes.

### Characterization of MurE and MurF within a single polypeptide

**Figure 5:**
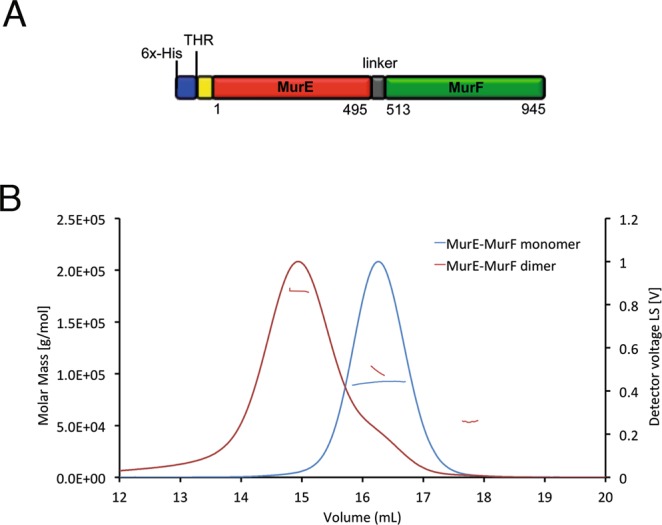
Characterization of MurE-MurF from *B. pertussis*. (**A**) Scheme of MurE-MurF employed in this study. (**B**) SEC-MALS chromatograms of MurE-MurF monomers and dimers, showing readings from the light scattering detector. The discontinuous lines indicate the calculated molecular masses of the two species.

In *B. pertussis*, the *murE* and *murF* genes are fused into a single transcript. This type of arrangement is unusual, since *murE* and *murF* occur mostly as individual genes (despite the fact that their position in the operon is highly conserved). However, other *Bordetella* spp., such as *B. avium*, *B. bronchiseptica*, *B. parapertussis*, and *B. petrii*, as well as *Pseudovibrio* spp. and some species of archaea (*Desulfobulbus* and *Desulfobacula* spp.) also carry the two genes in a single transcript. This organization yields a bifunctional MurE-MurF, which is a 945-residue protein with MurE and MurF being interconnected by a linker of approximately 20 residues (Fig. 5A). In order to characterize this connected MurE-MurF ligase form, we expressed the *B. pertussis* polypeptide in *E. coli* as a recombinant His-tagged protein.

Initial size exclusion chromatography (SEC) analyses led to the identification of two distinct forms of MurE-MurF that were pooled separately and further characterized by SEC-MALS (multi-angle laser light scattering^26^; Fig. 5B). Samples were injected on a Superdex 200 column coupled to an HPLC system and eluted in a buffer containing 25 mM HEPES pH 7.5, 300 mM NaCl, 10 mM MgCl_2_, and 5% glycerol. The system was coupled to UV-Vis, static and dynamic light scattering, and refractive index detectors in order to evaluate the absolute molecular mass and the hydrodynamic radius (Rh) of the species. The SEC-MALS profiles (Fig. 5B) revealed, for the largest form, a main maximum peak at 14.9 ml, for which a molecular mass of 179.5 kDa was experimentally determined. This value is close to that of a dimer, whose theoretical mass is 202.8 kDa (Rh of 5.5 nm). In the case of the smallest form, a peak was observed at 16.2 ml, with an experimental molar mass of 91.6 kDa. This value corresponds to the mass of a monomer, whose theoretical mass is 101.4 kDa (Rh of 3.5 nm; Table 2).

**Table 2:** Size and shape-related parameters obtained by SEC-MALS, SAXS and AUC.

Method	SEC-MALS		SAXS			SEC-MALS-SAXS	AUC
			Pair distribution				
Parameter	Rh (nm)	*f/f*_min_	Rg (nm)	Dmax (nm)	Vp (nm^3^)	ρ	*f/f*_min_
Monomer	3.5	1.22	4.1	14.6	151.9	1.1	1.42
Dimer	5.5	1.49	5.6	22.5	372.5	1.0	1.65

**Figure 6:**
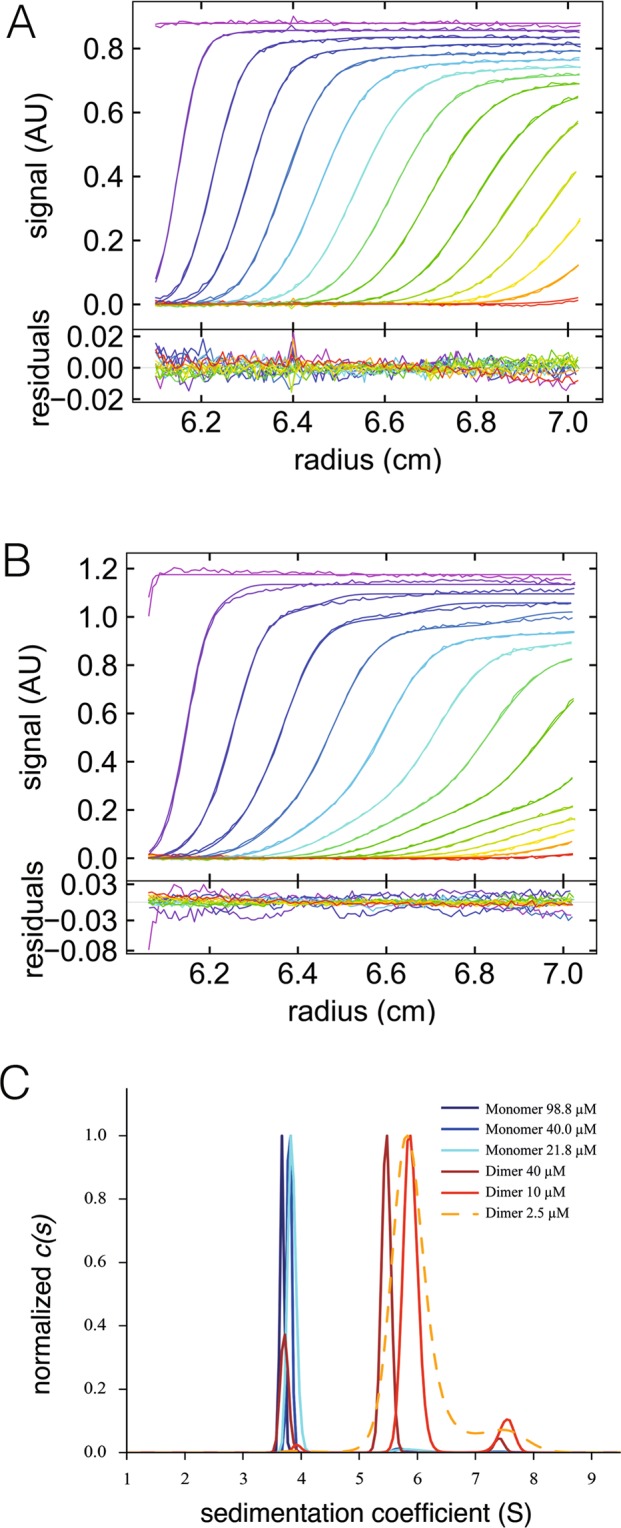
Sedimentation velocity AUC analyses of MurE-MurF from *B. pertussis*. Superposition of experimental and fitted sedimentation velocity AUC profiles obtained during 8 h of sedimentation at 42,000 rpm and 20 °C. Measurements were made at 280 nm, with a 3 mm optical path centerpiece, for monomeric (**A**) and dimeric (**B**) MurE-MurF at 40 µM. Residuals are shown in the lower panels. One of five acquired profiles is shown for clarity. (**C**) Superposition of the *c*(*s*) distribution profiles for the monomer and dimeric species of MurE-MurF, both having been tested at 3 different concentrations.

We sought to confirm these observations by performing sedimentation velocity AUC measurements. The experiments were performed in the same buffer, at 42,000 rpm using a Beckman ultracentrifuge at 20 °C with absorbance monitoring at 280 nm. Consecutive scans were automatically recorded at regular intervals (Fig. 6A,B). Dimeric MurE-MurF clearly sedimented faster than the monomeric form. The continuous size distribution *c(s)* analysis method in Sedfit was used to evaluate sample homogeneity, concentration effects, and to determine sedimentation coefficients.

For monomeric MurE-MurF, three different concentrations were tested: 99 μM (dark blue), 40 μM (blue) and 21.8 μM (cyan, Fig. 6C). AUC data for dimeric MurE-MurF were obtained at protein concentrations of 40 μM (dark red), 10 μM (red) and 2.5 μM (yellow) (Fig. 6C). For each of the two MurE-MurF forms, similar sedimentation profiles were obtained at all three concentrations, indicating that they do not undergo concentration-dependent association in these conditions. In the case of the dimer, the *s*-value for the main species decreases slightly when the concentration is increased, an effect related to hydrodynamic interactions that are governed by the frictional asymmetry of the particle, which affects the sedimentation process^27^. Measured values of *s* and *s*_*20,w*_ are reported in Table 3, and include the extrapolated *s*_*20,w*_ at infinite dilution, *s*_*20,w,0*_.

**Table 3:** Sedimentation velocity AUC results for the two species of MurE-MurF analyzed at 3 different concentrations.

MurE-MurF form	Conc. (μM)	*s* (S)	*s*_*20w*_ (S)	*s*_*20w,0*_ (S)	Theoretical mass (kDa)	Fitted mass (kDa)
monomer	98.8	3.67 +/− 0.06	5.15 +/− 0.10	5.45 +/− 0.1	101.4	113^a^; 83^b^
	40.0	3.79 +/− 0.05	5.30 +/− 0.08			
	21.8	3.84 +/− 0.05	5.40 +/− 0.08			
dimer	40.0	5.50 +/− 0.10	6.83 +/− 0.10	7.4 +/− 0.1	202.8	176^b^
	10.0	5.90 +/−0.10	7.40 +/− 0.10			
	2.5	5.90 +/− 0.10	7.40 +/− 0.10			

The *s*-value corresponds to the main species; *s*_*20,w*_ and *s*_*20,w, 0*_ are the *s*-values transformed to standard conditions (water at 20 °C), and at infinite dilution. Fitted molar masses were obtained for the monomer, from the non-interacting species analysis (^a^), and, for both, combining *s*_*20,w, 0*_ from AUC and Rh from SEC-MALS (^b^).

Monomeric MurE-MurF is homogeneous to 95%, which allows the AUC data to be fitted in terms of molar mass, without any assumptions. We found a molar mass of 113 kDa at the minimum concentration, close to the theoretical value of 101 kDa. This analysis was not applicable for the dimeric form of MurE-MurF, for which the main peak was only 80% of the sample. Combining the sedimentation coefficients and Rh allowed us to calculate experimental estimates of the molar masses in reasonable agreement with the theoretical values for the monomer and dimer (Table 3), and, considering the theoretical masses, estimates for the frictional ratio *f*/*f*_min_ (Table 2). The *f*/*f*_min_ estimates were slightly larger than those derived from Rh, but nevertheless confirmed that the dimer is more elongated as compared to the monomer. The dimeric MurE-MurF sample showed additional minor contributions (≈20%), consisting of monomers, which could be indicative of dissociation events, as well as larger species at or above 7.4 S (*s*_*20,w*_ = 9.3 S, for ~3 = 10%), which could correspond to larger aggregates.

### MurE-MurF is an elongated molecule with distant active sites

**Figure 7:**
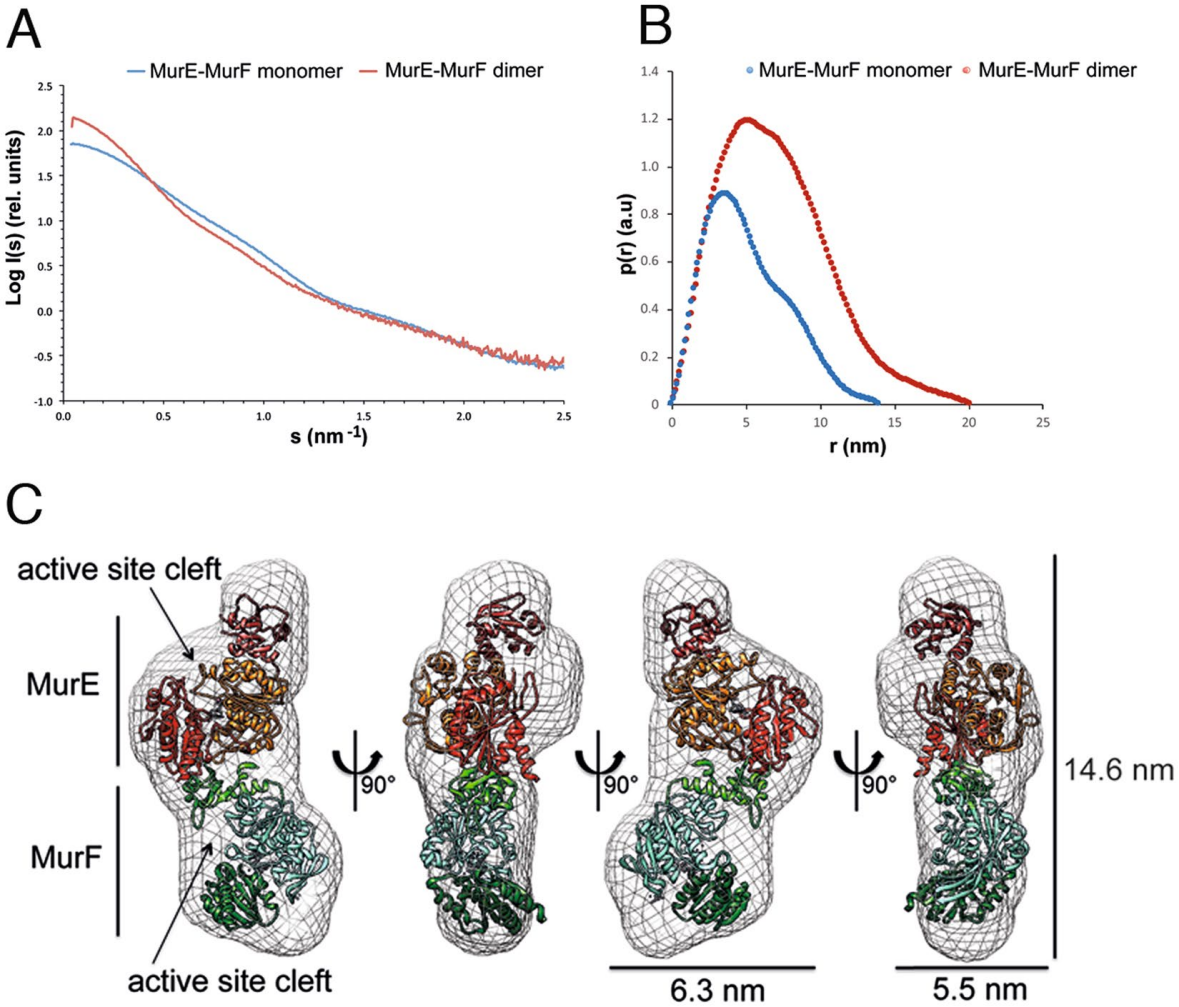
SAXS analysis of monomeric and dimer MurE-MurF. (**A**) The radially averaged scattered X-ray intensity (I) was plotted as function of the scattering angle (s). Scattering patterns for MurE-MurF monomer and dimer (blue and red, respectively) were recorded at different concentrations, but the two curves are related to the highest concentrations only (**B**) Plot of pair-distance distribution function, p(r), for the MurE-MurF monomer (light blue) and dimer (red). (**C**) SAXS envelope of the MurE-MurF monomer. The individual crystal structures of MurE and MurF from *T. maritima* (PDB codes: 4BUB and 3ZL8) (approx. 30% identity) were fitted using CHIMERA.

In order to understand how the MurE and MurF are structurally associated within the fused polypeptide, we performed small angle scattering (SAXS) experiments with both monomeric and dimeric forms. Scattering data were collected on beamline BM29 at the ESRF synchrotron in Grenoble. Scattering patterns were recorded at different concentrations (for both samples) and the data were plotted in the form of I(s) versus s (nm^−1^) with PRIMUS^28^. The data did not show any oligomerization or aggregation events since the normalized plots obtained at the different concentrations were superimposable (for each sample). Figure 7A shows the merged scattered data obtained from experiments performed with the two highest concentrations for both monomer and dimer. Scattering data were used to generate the pair distribution plots p(r) (for both monomer and dimer) using GNOM^29^ (Fig. 7B), that provided information on the position of electrons within the scattering samples. The real space gyration radius (Rg) and the Dmax were determined from the p(r) curves of the MurE-MurF monomer and dimer (Fig. 7B and Table 2)^30^.

Calculation of the shape factor ρ (Rg/Rh) for both samples yielded a value ≈ 1. Since globular proteins generally present ρ values in the range of 0.5–0.7^31^ these data indicate that both samples are elongated. This observation was supported by the *f/f*_min_ values calculated from the AUC data, which were in the range of 1.4–1.6 and thus indicated that both samples display an elongated shape. Shape and size-related parameters are reported in Table 2.

We employed our SAXS scattering data to build *ab initio* models of the two MurE-MurF forms using DAMMIF^32^ with default options. Ten different models were generated and averaged with DAMAVER^33^. Figure 7C shows the envelope of the MurE-MurF monomer visualized using CHIMERA^34^ and shown in 4 different orientations. The envelope confirmed the elongated shape of the polypeptide and it was subsequently used to fit the crystallographic models of the single MurE and MurF forms from *T. maritima*^18^, using CHIMERA. As a constraint to fit the two individual proteins into the SAXS envelope, we imposed the C-terminus of MurE to be in close proximity to the N-terminus of MurF. We then approximately positioned the two structures in the SAXS envelope, and ran the “fit in Map” option of CHIMERA using the map simulated from atoms at 15 Å resolution for both structures. The best fit of the two individual proteins into the SAXS envelope suggests a head-to-tail arrangement in which the C-terminus of MurE is in close proximity to the N-terminus of MurF. In this arrangement, the active sites are distant from each other (≈35 Å). The minimized value of χ^2^ that compares the theoretical data from the PDB coordinates of the fused MurE-MurF fitted structure to the MurE-MurF scattering curve, calculated by using CRYSOL is 6.9 (theoretical optimal fit = 1.0)^35^. This observation suggests that MurE-MurF could contain flexible regions that cannot be well fitted using the rigid crystallographic models. Nevertheless, these observations indicate how this elongated, flexible arrangement could favor interactions with other Mur enzymes within a higher order complex, as discussed below.

Attempts to fit individual MurE and MurF X-ray models into an envelope of the MurE-MurF dimer calculated with CHIMERA yielded numerous possible different arrangements that prevented further conclusions. This could be due to the fact that the MurE-MurF dimer displays different conformations, as noted above, and the SAXS data would thus represent an average of different forms that are present in solution^36^. We thus sought to obtain further information on the shape of dimeric MurE-MurF by employing negative stain EM. Homogeneous, gel-filtered samples of the MurE-MurF dimer were imaged, allowing selection of circa 40,000 individual frames subjected to 2-D classification (Fig. 4B). Many class averages are well defined and elongated, and in several cases, four distinct regions, which could represent individual subdomains, can be clearly identified. Class-averages manifesting C-, L- or S-like forms indicate flexibility and a potential mixture of conformational states.

### MurF is more active in the monomeric than in the dimeric MurE-MurF polypeptide

**Figure 8:**
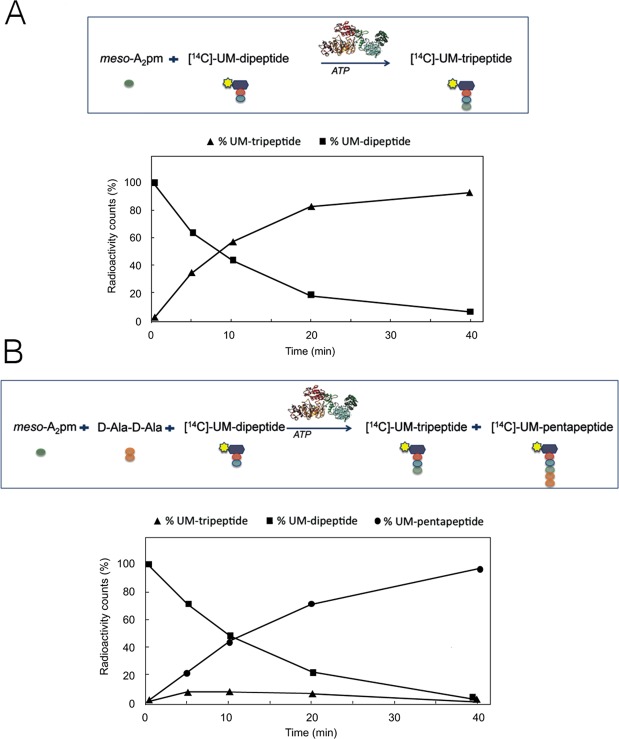
MurE-MurF is catalytically active. (**A**) The *meso*-A_2_pm-adding activity (MurE) of the MurE-MurF fusion protein was tested individually using ATP, *meso*-A_2_pm and UM-[^14^C]dipeptide as substrates. The kinetics of consumption of the latter radiolabeled substrate (■) and formation of the UM-[^14^C]tripeptide product (▲) were followed as described in Materials and Methods. (**B**) The same assay mixture was supplemented with D-Ala-D-Ala so that the intermediate UM-tripeptide MurE product could subsequently be used by MurF to generate radiolabeled UM-pentapeptide (MurE-MurF coupled assay). The respective amounts of the UM-dipeptide substrate and of the intermediate (UM-tripeptide) and final (UM-pentapeptide) reaction products (■, ▲ and ●, respectively) were determined at different times, as detailed in the text.

In order to understand the potential importance of dimerization of the MurE-MurF polypeptide, we measured MurE and MurF activities of the monomeric and dimeric forms of MurE-MurF. The *meso*-A_2_pm-adding and D-Ala-D-Ala-adding activities, which correspond to MurE and MurF-catalyzed reactions, were first tested individually. The activity of MurE was tested by following the amount of radiolabeled UM-[^14^C]dipeptide that was consumed and of UM[^14^C]tripeptide formed in time (Fig. 8A), while the activity of MurF was assessed by measuring the amount of UM[^14^C]tripeptide that was consumed and UM[^14^C]pentapeptide formed. The measurements were performed four times and yielded very similar results (variations < 10%). One set of representative results is shown here. In the standard assay conditions used, the corresponding specific activities of MurE and MurF were estimated at 6.2 and 14.1 µmol/min/mg for the monomer, and 5.6 and 7.5 µmol/min/mg for the dimer, respectively. This indicates that MurF is twice as active in the monomeric form of the protein than in the dimeric one.

Notably, there was no increase in the overall rate of product formation when a MurE-MurF coupled assay was used (Fig. 8B). The amount of intermediate product UM-tripeptide remained relatively low in these conditions, being rapidly transformed to UM-pentapeptide by MurF. These data showed that the two protein activities are independently functional, that the MurF activity is not limiting as compared to MurE (at least *in vitro* and in the assay conditions used), and that the coupling of their activities does not provide any apparent advantage in terms of catalytic efficiency. However, the fact that the specific activity of the MurF-catalyzed reaction in the monomer is twice that of the dimer is noteworthy, since it could point to a regulatory mechanism where dimerization decreases catalytic rates. The interaction between protein subunits is essential for regulation and catalysis in several complex systems^37^, and it is conceivable that oligomerization of Mur ligases could play a role in regulation of enzyme activity and channeling of intermediates towards later steps in the pathway.

### MurE-MurF interacts with MurG with a micromolar K_D_

**Figure 9:**
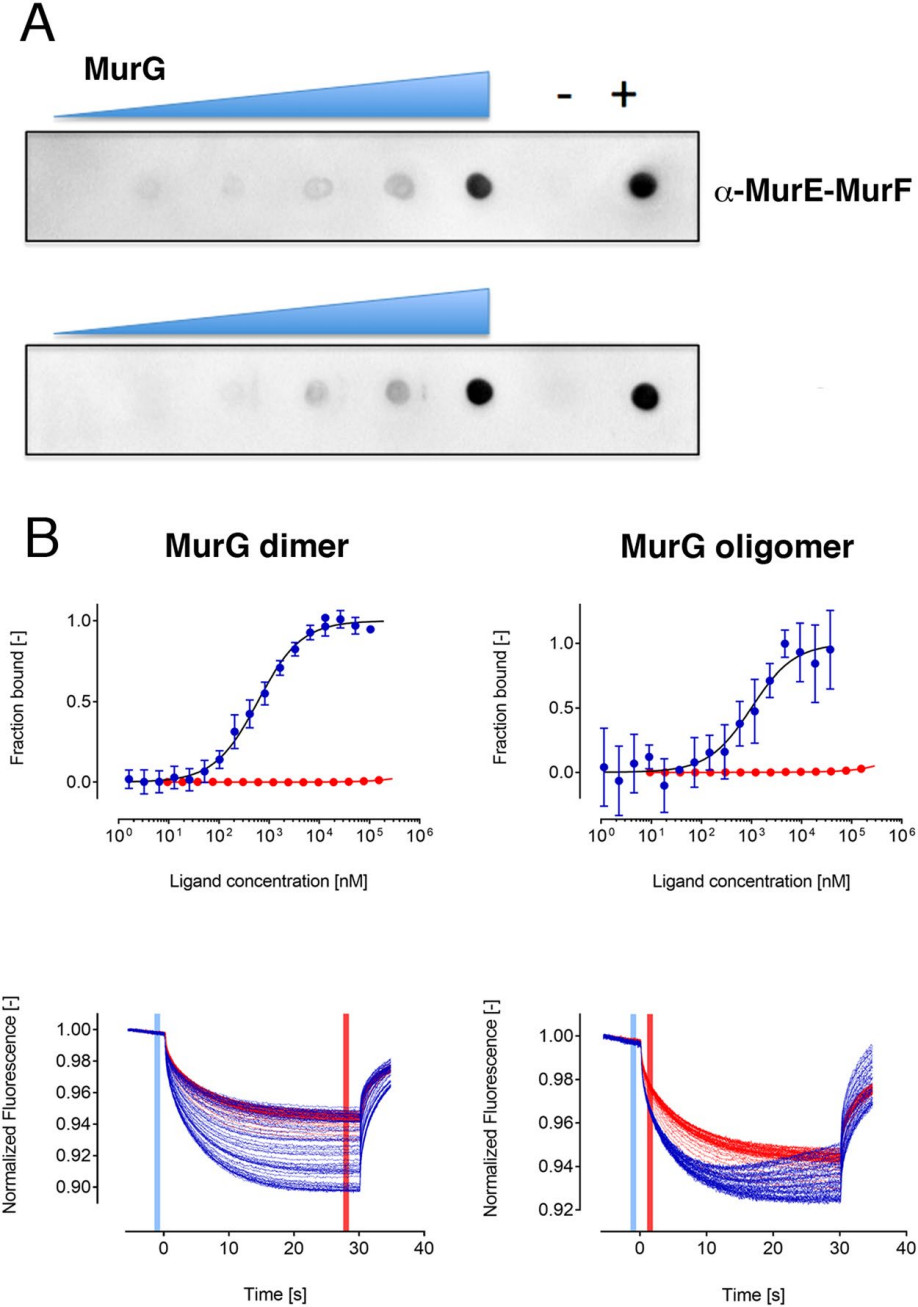
MurE-MurF interacts with both forms of MurG. (**A**) Dot Blot assays performed on a nitrocellulose membrane where MurG was blotted in increasing concentrations and subsequently incubated with a solution of MurE-MurF. Top, Dimeric MurG, purified in detergent; Bottom, oligomeric MurG, purified in its absence. Positive control: purified MurE-MurF. Negative control: an unrelated bacterial protein. (**B**) MST binding curves were fitted to derive the K_D_ between MurG and MurE-MurF (in blue) in the presence (left) or absence (right) of DM. The curves related to the experimental control (LAMTOR4/5, a eukaryotic protein from the mTor system, unrelated to peptidoglycan biosynthesis) are in red. Error bars = s.d; n = 3. Solid lines represent linear extrapolations. Bottom: MST traces corresponding to the titration of MurG (in blue) and the control protein (in red) against 50 nM of MurE-MurF-FITC in the presence and absence of DM, respectively.

Having characterized MurE-MurF and MurG independently, we set out to analyze their potential interaction in order to obtain further evidence of the existence of a Mur ligase complex. Initially, we performed a Dot Blot experiment by loading MurG purified both in the presence or absence of detergent on a membrane and incubating with MurE-MurF. Development of the membrane with anti-MurE-MurF antibodies (Fig. 9A) indicated a concentration-dependent interaction between the proteins in both cases. Subsequently, we performed microscale thermophoresis (MST) using fluorescein isothiocyanate (FITC)-labeled MurE-MurF at a final concentration of 50 µM. Labeled MurE-MurF was mixed with serial concentrations of unlabeled MurG (from 0.3 nM to 2.6 μM) in a buffer containing 25 mM HEPES pH 7.5, 150 mM NaCl, 10 mM MgCl_2_, 5% glycerol and 0.17% w/v DM. Regarding oligomeric MurG, the sample was purified in the absence of detergent and experiments were performed using concentrations of MurG ranging from 1 nM to 37 μM. All samples were loaded into MST standard capillaries and mounted on a NanoTemper Monolith apparatus.

Figure 9B (left) displays the binding curves corresponding to the experiment between labeled MurE-MurF and MurG (purified in the presence of detergent). This shows a dose-dependent response that allowed the calculation of a K_D_ value of 0.57 ± 0.05 μM. In the case of the experiment performed with oligomeric MurG (Fig. 9B, right) the dose-dependent curves did not allow the attainment of a clear plateau, possibly due to the fact that the MurG sample is not composed of a single oligomeric species. However, we were able to measure an apparent K_D_ value of 0.97 ± 0.25 μM, that is in the range of the K_D_ obtained for dimeric MurG. This indicates that both forms of MurG can interact with MurE-MurF, providing further evidence of the existence of a multi-Mur complex in the bacterial cytoplasm.

## Discussion

Bacterial morphogenesis is a process that is closely linked to peptidoglycan biosynthesis. Lipid II, the basic building block of peptidoglycan, is synthesized in the bacterial cytoplasm through the action of Mur enzymes A-F, as well as MraY and MurG^38^. Since Lipid II is required for both cell wall elongation and division, these enzymes are essential for the functioning of both the elongasome and the divisome. The detection of interactions between Mur ligases and elongasome/divisome partners, including MurF:MraY in *C. crescentus*, MurF:MreB in *C. pneumoniae*, and MurD/MurE/MurF with both MreB and MurG in *T. maritima*, underlines the importance that Murs have in both processes^15,17,18,39^. These associations, that could occur within a multi-membered complex on the boundary between cytoplasm and inner membrane, could facilitate the transport of peptidoglycan building blocks towards the inner leaflet of the membrane^12,15,16^. MurG, an essential enzyme^40^ that interacts and/or co-localizes with MreB, RodA, MraY, MurD, MurE, and MurF in different bacteria^15,17,18,39^ has been suggested as playing the role of scaffold within this potential Mur ligase/peptidoglycan biosynthesis complex. MurG is peripherally associated to regions of increased fluidity (RIFs) within the inner leaflet of the plasma membrane; treatment of cells with the antibiotic daptomycin detaches MurG from RIFs, which is enough to block cell wall synthesis^20^.

Biochemical and structural studies of MurG from *E. coli* have shown that it can form either monomers or dimers^21,22,25,41^. However, the crystals used to solve the structures were obtained in the presence of detergents, which may not emulate the cell wall environment appropriately. Our results indicate that MurG can exist as higher order species, both as a purified molecule and when associated to bacterial membranes, being able to form tetramers as well as oligomers of higher stoichiometry that interact with MurE-MurF with comparable affinities. This suggests that a higher order oligomer could also be one of the biologically active forms of MurG, with monomeric and/or dimeric variants representing its ‘building blocks’. It is of note that glycosyltransferases working in different pathways have also been shown to form higher order oligomers in order to associate with partner molecules^42,43^. A scaffolding role for MurG, involving the recognition of multiple partners could be facilitated through oligomerization.

MurG has been shown to be essential for both cylindrical and polar peptidoglycan synthesis. Localization of MurG using immunofluorescence in *E. coli* cells showed that the protein forms discrete membrane-associated foci. Ribosome profiling experiments have indicated that there are approximately 518 copies of MurG/cell^44^; this suggests that each one of the foci could contain between 10 and 25 MurG molecules^17^. MurG has also been shown to accumulate near midcell in FtsZ-dependent fashion^45^. These observations suggest that the last step of Lipid II biosynthesis occurs in specific sites of the cell, where the concentration of MurG would be high enough for self-association, as shown here.

MurG has also been suggested as being part of subassemblies that could associate to other division^17^ or elongation proteins. Notably, White and coworkers^15^ described the co-localization of Mur ligases MurC, MurE, MurF, as well as MurB and MraY, with MurG in *C. crescentus*. These results were also supported by two-hybrid results that indicated interactions between MraY and both MurF and MurG. Hence, there is now growing evidence that Mur ligases do associate, potentially in subcomplexes, in different bacteria. The observation that MurE and MurF (as well as MurC and MurG) are transcribed from fused genes in a number of bacteria^12^ is indicative of the advantage that having these enzymes in close proximity, within the bacterial cytoplasm, can provide.

This is the case for MurE and MurF of *B. pertussis*, studied here. Their co-transcription from a single gene could have been indicative of a possibility that, once folded, they would form a globular structure with active sites in close proximity; however, our SAXS and EM results clearly show that MurE-MurF is an elongated, flexible polypeptide. It is of interest that crystal structures of individual Mur ligases have also indicated that these three-domain proteins display considerable flexibility, with C-terminal regions that are ‘free’ to test different conformations in three-dimensional state^18^. It is conceivable thus that Mur ligase flexibility may be a necessary element for association with different intracellular partners during either cell division or cell wall elongation, facilitating binding/release events in accordance with the peptidoglycan biosynthesis requirement of the cell cycle. A potential structural stabilization of the MurE-MurF bifunctional protein in the presence of ligands is still under investigation.

The spatial coupling of related metabolic reactions can be facilitated through controlled proximity. This can concentrate reactants in a specific region of the cell, enable the removal of inhibitory products, or channel molecules from one active site to the next^46^. Our biochemical and structural data describing the different oligomeric forms of MurG, coupled to our results revealing the flexibility of the bifunctional MurE-MurF and its capacity to interact with different forms of MurG suggest that within the bacterial cytoplasm, MurG oligomers could play the role of peptidoglycan complex scaffolds. This model provides an explanation as to how a protein with a molecular mass of 50 kDa can interact, often concomitantly, with a number of different partners, including soluble, membrane-bound, and filament-forming proteins. In the future, this possibility could be further tested with the employment of techniques such as Raster Image Correlation Spectroscopy (RICS), for example, in order to evaluate protein oligomerization on cellular membranes as well as in live cells^47^. This work thus provides an initial framework towards the understanding of cytoplasmic complexes that regulate and facilitate peptidoglycan biosynthesis during different phases of the cell cycle, which could lead the way to the eventual employment of Mur enzymes as *de facto* targets for antibiotic development.

## Materials and Methods

### Cloning and expression of MurE-MurF and MurG

The *murE-murF* gene from *B. pertussis* (strain ATCC 9797/DSM No 5571) was amplified using conventional PCR methods and cloned into a pET15b vector (Novagen) in frame with a thrombin-cleavable N-terminal 6-histidine tag. The *murG* gene from the same strain was cloned into a pET30b vector modified in order to express the protein with a TEV-cleavable N-terminal Strep-tag sequence.

*E. coli* RIL cells carrying the expression vector for the MurE-MurF fusion protein were grown at 37 °C in Luria-Bertani medium (LB), supplemented with 100 mg/l ampicillin and 34 mg/l chloramphenicol. Expression was induced by the addition of IPTG to a final concentration of 0.2 mM at OD_600nm_ = 0.7 A.U., and the cells were harvested by centrifugation (6,000 rpm, 20 min, 4 °C) after overnight growth at 16 °C.

*E. coli* BL21(DE3) cells carrying the MurG expression vector were grown at 37 °C in LB medium supplemented with 50 mg/l kanamycin. Over-expression was induced by the addition of IPTG to a final concentration of 1 mM at OD_600nm_ = 0.8 A.U., and the cells were collected by centrifugation (6,000 rpm, 20 min, 4 °C) after overnight growth at 18 °C.

### Purification of MurE-MurF

Cells were resuspended in buffer A (25 mM HEPES pH 7.5, 100 mM NaCl, 25 mM imidazole, 5 mM β-mercaptoethanol) supplemented with protease inhibitors cocktail (Sigma-Aldrich) and 200 μg/ml lysozyme (Fluka). Cell lysis was carried out by passing the sample three times through a pre-cooled cell disruptor (Constant Systems) at 15,000 psi. The soluble fraction was obtained by centrifugation at 18,000 rpm for 1 h at 4 °C and was loaded onto a pre-equilibrated 5 ml Ni-NTA column (Qiagen). After a washing step to eliminate the unbound proteins, the hexahistidine-tagged protein was eluted from the column with buffer B (25 mM HEPES pH 7.5, 100 mM NaCl, 500 mM imidazole, 5 mM β-mercaptoethanol). The fractions containing MurE-MurF were pooled and further purified by size-exclusion chromatography (SEC, Superdex 200, 10/30, GE) in buffer C (25 mM HEPES pH 7.5, 200 mM NaCl, 1 mM EDTA, 10 mM TCEP). The protein-containing fractions were collected, pooled and analyzed by SDS- PAGE. The protein was concentrated in a 50 kDa cut off concentrator (Vivaspin).

### Purification of MurG

Cells were resuspended in buffer D (25 mM HEPES pH 7.5, 300 mM NaCl, 10 mM MgCl_2_, 5% glycerol, 5 mM DTT) supplemented with 0.5% (w/v) DDM, a protease inhibitor cocktail (Sigma-Aldrich) and 200 μg/ml lysozyme (Fluka). Cell lysis was carried out by passing the sample four times through a precooled cell disruptor at 15,000 psi. Cell debris were removed by centrifugation at 18,000 rpm, for 1 h at 4 °C and the cleared lysate was loaded on pre-equilibrated 5 ml Strep-trap column (Qiagen). After a washing step in buffer D supplemented with 0.01% (w/v) DDM, the Strep-fused protein was eluted in buffer D supplemented with 0.01% (w/v) DDM and 2.5 mM d-desthiobiotin. Fractions containing purified MurG were analyzed by SDS-PAGE.

### Cross-linking of MurG on bacterial membranes

MurG was over-expressed in cells as described before and 4.4 g of cell pellet were re- suspended in ice-cold 25 mM HEPES pH 7.5. The sample was passed three times through a refrigerated cell disruptor at 15,000 psi. Unbroken cells and debris were removed by low-speed centrifugation (10,000 rpm, for 10 min at 4 °C). The supernatant was collected and the membrane portion pelleted by ultracentrifugation at 40,000 rpm for 1 h at 4 °C and re-suspended in 25 mM HEPES pH 7.5. 100 μl of the re-suspended membranes were incubated with increasing concentrations of DMP (from 0.1 to 5 mM) and incubated for 30 min at room temperature. Cross-linked samples were quenched by addition of 50 mM Tris-HCl pH 8.0 and were loaded on a SDS-PAGE gradient gel (4–20%).

In order to detect MurG by Western blotting, samples were transferred to a nitrocellulose membrane for 1.5 h at a constant voltage of 300 V. Following protein transfer, the membrane was blocked for one hour with shaking at room temperature in 3% BSA PBS-T (PBS-Tween 0.05%). The membranes were incubated overnight at 4 °C with shaking with anti-MurG primary antibodies (mouse, by Covalab) at a dilution of 1:5,000. Lastly, they were incubated with secondary antibodies (anti-mouse) for 1 h at room temperature. The signal was developed using SIGMA FASTDAB tablets with Metal Enhancer according to manufacturer’s instructions.

### Cross-linking of purified MurG

Cross-linking experiments were performed with EGS (ethylene glycol bis(succinimidyl succinate); Thermo Scientific) solubilized in DMSO. MurG was prepared in buffer containing HEPES pH 7.5, 200 mM NaCl and 0.11% DM and mixed with increasing concentrations of EGS (0.1, 1.0, 2.5 mM) in a final volume of 100 μl. The reaction was incubated at room temperature for 30 min and afterwards quenched by adding Tris-HCl buffer pH 8.0 at a final concentration of 50 mM. The reaction was incubated for additional 15 min and 15 μl of the reaction were loaded on a SDS-PAGE gradient gel (4–20%).

### Size exclusion chromatography analysis of MurG

MurG was extracted from the bacterial membrane and purified by affinity chromatography as described above; subsequently, buffer exchange was carried out on a Superose 6 10/300 column (GE). The column was pre-equilibrated, before sample injection, with buffer C either in the absence or in the presence of detergents (DDM and DM) at a concentration corresponding to 1.2 x CMC. 500 μl of sample at a concentration of 2 mg/ml were centrifuged at 13,000 rpm for 20 min at 4 °C and injected onto the column. The sample was eluted at a flow rate of 0.5 ml/min at 4 °C.

### Analytical ultracentrifugation (AUC)

The experiment was performed at the biophysics characterization platform of the ISBG, Grenoble. Concentrated samples were loaded onto 3 mm 2-channel Ti centerpiece cells (100 μl), while low concentration samples were loaded onto 12 mm 2-channel Ti centerpiece cells (400 μl; Nanolytics, Potsdam, Germany). Cells were filled with buffer without detergent (25 mM HEPES pH 7.5, 500 mM NaCl, 10 mM MgCl_2_, 5% glycerol) in the reference sector and with sample solution in the sample sector. Sedimentation velocity experiments were performed using a XLI analytical ultracentrifuge and an Anti-50 rotor (Beckman Coulter, Palo Alto, USA) at 42,000 rpm and 20 °C. Two optical systems were used for the observation of sedimentation: absorbance (280 nm and eventually 250 nm) and interference (655 nm). Data were processed with Redate 1.0.1 (http://biophysics.swmed.edu/MBR/software.html) prior to being analyzed with SEDFIT v 15.01b (https://sedfitsedphat.nibib.nih.gov/software/)^48^ and GUSSI 1.2.1 (http://biophysics.swmed.edu/MBR/software.html)^49^. We employed the continuous size distribution *c*(*s*) analysis method to determine the values of the sedimentation coefficients, *s*. Sedimentation coefficients were corrected to standard conditions as *s*_*20,w*_, taking into consideration, for both MurG and MurE-MurF, partial specific volumes calculated from the sequences in Sedfit. These values corresponded to 0.744 and 0.734 ml/g, respectively. For MurG tested in the presence of detergent, a mean partial specific volume between that of the protein and DDM (0.82 ml/g^50^) or DM (0.79 ml/g calculated from^51^) was employed. Solution density (*ρ* = 1.036 g/ml) and viscosity (*η* = 1.259 cp) values were measured experimentally at 20 °C on an Anton Paar DMA 5000 density meter and an Anton Paar AMVn rolling ball viscometer, respectively. Putative oligomeric states were derived from *s* considering the Svedberg equation:

$$s=\frac{M(1-\rho \overline{{\rm{v}}})}{{N}_{A}6\pi \eta Rh}$$

where *N*_A_ is Avogadro’s number. *M* and $\overline{v}$ are the molar mass and the partial specific volume of the assemblies, which depend, for MurG in detergent, on bound detergent, δ. δ was estimated from the integration of peaks in the *c*(*s*) distributions obtained at 280 nm and with interference; Rh is the hydrodynamic radius, and is related to R_min_, the radius of the anhydrous volume ($\overline{v}$*M/N*_A_), and to the frictional ratio *f*/*f*_min_ = Rh/R_min_^52^.

### SEC-MALS (multi-angle laser light scattering)

The experiment was performed at the biophysical analysis platform of the ISBG, Grenoble. A Shimadzu Prominence HPLC system equipped with a Superose 6 SEC column was connected in line with a mini DAWN-TREOS and a DynaPro NANOSTAR static and dynamic light scattering instrument (Wyatt Technologies, Santa-Barbara, USA), an Optilab rEX refractometer (Wyatt Technologies) and a UV-Vis detector SPD-M20A. The light scattering and refractive index detectors were calibrated following the manufacturer’s instructions. The SEC column was equilibrated with buffer C and 45–100 μl of each sample were injected with a flow rate of 0.5 ml/min. Data from the three detectors were imported by the Astra V software version 5.4.3.20 (Wyatt Technologies) and processed following the manufacturer’s guidelines. The experiment was performed at room temperature. For the analysis of MurE-MurF the ΔLS and the ΔRI signals were collected and the experimental mass was derived from derived from the calculated from the sequence dn/dc value of 0.185 ml/g for MurE-MurF.

### Small-angle X-ray Scattering (SAXS)

SAXS measurements were recorded at the BM29 beamline of the European Synchrotron Radiation Facility (Grenoble, France). Prior to data collection, a control experiment was conducted recording the scattering curve of BSA at 5 mg/ml. For the MurE-MurF monomer the experiments were performed at 1, 3, 5, 7 and 10 mg/ml; for the MurE- MurF dimer at 0.5, 1, 3 and 5.6 mg/ml. Scattering data from a buffer solution were recorded and subsequently subtracted from the MurE-MurF sample scattering curves. No radiation damage was observed during the 10 s radiation exposure frame and all the data were collected at 20 °C. Data analysis were carried out by following default parameters of the PRIMUS software package^28^. The radius of gyration (Rg) and the forward scattering value I(0) were estimated using the Guinier approximation^53^. Both parameters, as well the maximum particle dimension Dmax, were calculated by using GNOM^29^. *Ab initio* models were generated using DAMMIF^32^. A final averaged model was generated using 10 independent models using DAMAVER^33^. The fitting of molecules was performed using the FIT INTO MAP command of CHIMERA^54^ and the χ^2^ value measured using Crysol^35^.

### Enzymatic assays

#### Materials

UM-L-Ala-D-Glu, UM-L-Ala-γ-D-Glu-*meso*-A_2_pm, UM-L-Ala-D-[^14 ^C]Glu and UM-L-[^14 ^C]Ala-γ-D-Glu-*meso*-A_2_pm were prepared according to published procedures^10^.

#### MurE assay

The standard MurE activity assay measured the formation of radiolabeled UM-L-Ala-γ-D-Glu-*meso*-A_2_pm in a reaction mixture (40 μl) containing 100 mM Tris-HCl pH 8.6, 20 mM MgCl_2_, 5 mM ATP, 0.1 mM UM-L-Ala-D-[^14 ^C]Glu (400 Bq), 0.2 mM *meso*-A_2_pm, and pure enzyme (20 μl of an appropriate dilution in buffer E (20 mM potassium phosphate, pH 7.2, 1 mM DTT)).

#### MurF assay

The standard MurF activity assay measured the formation of radiolabeled UM-L-Ala-γ-D-Glu-*meso*-A_2_pm-D-Ala-D-Ala in a reaction mixture (40 μl) containing 100 mM Tris-HCl pH 8.6, 20 mM MgCl_2_, 5 mM ATP, 0.1 mM UM-L-[^14 ^C]Ala-γ-D-Glu-*meso*-A_2_pm (400 Bq), 0.2 mM D-Ala-D-Ala, and pure enzyme (20 μl of an appropriate dilution in buffer E).

#### Coupled MurE-MurF assay

In this assay that measured the formation of the two radiolabeled products UM-tripeptide and UM-pentapeptide, the reaction mixture (40 μl) contained 100 mM Tris-HCl pH 8.6, 20 mM MgCl_2_, 5 mM ATP, 0.1 mM UM-L-Ala-D-[^14 ^C]Glu (400 Bq), 0.2 mM *meso*-A_2_pm, 0.2 mM D-Ala-D-Ala, and pure enzyme (20 μl of an appropriate dilution in buffer E). After incubation for 40 min (5 to 40 min in some assays) at 37 °C, these reactions were terminated by the addition of glacial acetic acid (8 μl), followed by lyophilization. In all cases, the radioactive substrates and products were separated on a Nucleosil 100 C18 5U column (150 × 4.6 mm; Alltech France) using 50 mM ammonium formate pH 4.0, at a flow rate of 0.6 ml/min. Retention times of UM-dipeptide, UM-tripeptide and UM-pentapeptide were 18, 9.5, and 32 min in these conditions, respectively. Radioactivity was detected with a flow detector (model LB506- C1; Berthold) using the Quicksafe Flow 2 scintillator (Zinsser Analytic) at 0.6 ml/min and quantification was performed with the Radiostar software (Berthold).

### Dot Blot assay

MurG purified either in the presence of 1.2 x CMC DDM or in its absence was spotted onto a nitrocellulose membrane at different concentrations (from 50 ng to 1 μg) in a final volume of 2 μl. The positive and the negative control samples were spotted at 0.8 μg. After being allowed to dry for 30 min, the membrane was blocked in PBS-Tween 0.05% (PBS-T) supplemented with 1% Bovine Serum Albumin (BSA) for 1 h at room temperature with agitation. After one washing step in PBS-T, the membrane was incubated overnight at 4 °C in a PBS-T solution containing 0.1 mg/mL of MurE-MurF. Subsequently, the membrane was stained with anti-MurE-MurF primary antibodies at a dilution of 1:500 overnight at 4 °C then incubated with anti-rabbit secondary antibodies for 1 h at room temperature. Detection was performed with SIGMA FASTTM DAB tablets with Metal Enhancer.

### Microscale Thermophoresis (MST)

MurE-MurF was incubated overnight with a 3-fold molar excess of FITC in labelling buffer (25 mM HEPES pH 7.5, 150 mM NaCl, 10 mM MgCl_2_ and glycerol 5%), in darkness and at 4 °C. Excess FITC was removed by using a PD-10 desalting column and fractions containing the FITC labeled MurE-MurF were pooled and used for the assay. Protein concentration was quantified by measuring the absorbance at 280 nm using a Nanodrop. For the experiment with monomeric MurG, a fixed concentration of labeled MurE-MurF (50 µM) was mixed with serial/increasing concentrations of purified MurG ranging from 0.3 nM to 26 µM in a final buffer containing 25 mM HEPES pH 7.5, 150 mM NaCl, 10 mM MgCl_2_, 5% glycerol and 0.17% w/v *n*-decyl-β-D-maltopyranoside (DM). The samples were inserted into MST standard capillaries and measurements were performed using a Nano Temper Monolith NT.115 instrument equipped with a blue-red detector. Detection of labeled protein was performed using the blue LED-filter (excitation at 495 nM) at 22 °C, 20% MST power and 20% LED intensity. On and Off laser times were set at 5 and 25 s, respectively. Each sample was measured in triplicate. Data sets were processed and analyzed using the MO affinity software version 2.1 (NanoTemper Technologies, Munich, Germany). The relative normalized fluorescence, ΔF_norm_ (‰), defined as F_norm hot_/F_norm cold_, was plotted against analyte concentration to generate the binding curve. In the control experiment, labeled MurE-MurF was incubated in increasing concentrations of an unrelated eukaryotic protein.

The experiment with the oligomeric form of MurG was performed using a fixed concentration of labeled MurE-MurF (50 µM), which was mixed with serial/increasing concentrations of purified MurG ranging from 1 nM to 37 µM in a final buffer containing 25 mM HEPES pH 7.5, 150 mM NaCl, 10 mM MgCl_2_ and 5% glycerol. The samples were inserted into MST Premium capillaries and measurements were performed using a Nano Temper Monolith NT.115 instrument equipped with a blue-red detector.

### Grafix and electron microscopy

In order to stabilize oligomeric MurG for negative stain electron microscopy, GraFix was carried out on purified MurG^24^. 50 μl of sample at a concentration of 1.6 mg/ml were applied to the top of a 1 ml centrifuge tube (Beckman) containing 10–30% (v/v) glycerol and 0–0.15% (v/v) glutaraldehyde gradient prepared in 25 mM HEPES pH 7.5 and 300 mM NaCl. Gradients were ultracentrifuged for 14 h at 50,000 rpm and 4 °C. Following centrifugation, the gradient was fractionated from the top of the ultracentrifugation tube and the single 25 μl fractions were transferred to a 96-well plate. Fractions were tested using a Bradford assay and subsequently analyzed by negative stain EM in order to detect the most homogeneous sample.

3 μl of sample were applied to the clean side of carbon on a carbon–mica interface and stained with 2% sodium silicotungstate. Micrographs were recorded on a FEI Tecnai T12 microscope operated at 120 kV with a Gatan Orius 1000 camera. Images were recorded at a nominal magnification of 29,000 x resulting in a pixel size of 2.22 Å.

Semi-automatic particle selection was carried out with BOXER^55^, with box sizes of 112 × 112 pixels for MurG and 82 × 82 pixels for MurE-MurF. After initial cleaning, a total of 13,831 particles were extracted for MurG and 40,867 for MurE-MurF. CTF correction and several rounds of 2D reference-free classification were then performed in RELION^56^.
